# Pupil Wizard: an app to enhance knowledge of pupillary abnormalities

**DOI:** 10.1038/s41433-025-03763-9

**Published:** 2025-03-25

**Authors:** Øystein Kalsnes Jørstad, Andrew G. Lee

**Affiliations:** 1 Department of Ophthalmology, Oslo University Hospital, Oslo, Norway; 2 Faculty of Medicine, University of Oslo, Oslo, Norway; 3 The Houston Methodist Research Institute, Houston Methodist Hospital, Houston, TX USA; 4 Departments of Ophthalmology, Neurology, and Neurosurgery, Weill Cornell Medicine, New York, NY USA; 5 Department of Ophthalmology, University of Texas Medical Branch, Galveston, TX USA; 6 University of Texas MD Anderson Cancer Center, Houston, TX USA; 7 Texas A&M College of Medicine, Houston, TX USA; 8 Department of Ophthalmology, The University of Iowa Hospitals and Clinics, Iowa City, IA USA

**Keywords:** Eye manifestations, Pupil disorders

## To the Editor:

The pupils serve as a window to the brain, as their responses provide valuable insights into brain function, neurological integrity, and autonomic nervous system activity. The ability to accurately identify pupillary findings is essential in clinical practice; some findings, such as physiological anisocoria, are entirely harmless, whereas others, such as mydriasis in compressive third-nerve palsy, may indicate life-threatening conditions that require immediate management.

A pedagogical challenge in teaching and learning of pupillary disorders is their highly dynamic nature, with varying reactions to light stimuli, near focus, and pharmacological agents. There are several comprehensive articles on diagnosing pupillary abnormalities, but passively learning pupil testing through static illustrations and text remains challenging [1–4]. Moreover, many pupillary disorders are relatively uncommon (for instance, even a fully trained physician may never have encountered a case of Argyll Robertson pupil), making it difficult to gain broad clinical experience within a student semester or residency. An alternative is to watch educational videos, but this remains a passive learning process. In contrast, there is a general support in the literature for active learning in medical education, as it is effective in engaging students, among other benefits [5].

**Fig. 1:**
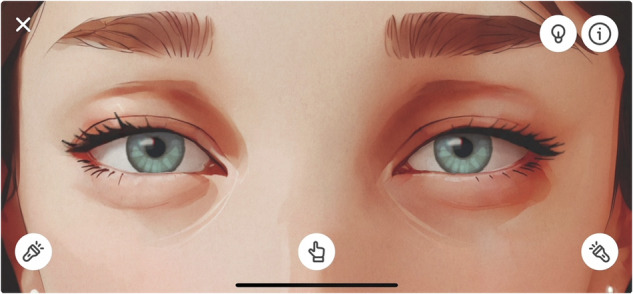
The figure shows a screenshot of the Eye Wizard app. The symbols represent the active elements on the screen: the penlights test the pupillary light reflexes, the finger tests the accommodation-convergence reflex, the light bulb toggles the ambient light, and the information symbol provides details about the condition.

As neuro-ophthalmology educators, we have sought ways to improve the teaching of pupil-related disorder, focusing on incorporating their dynamic aspects and active learning. Our solution is an app for smartphone and tablet devices. The app, Pupil Wizard, provides a digital textbook featuring a dynamic presentation of the key pupillary abnormalities. It allows the users to interact with a digital patient and explore how each condition responds to direct and indirect light stimuli, near focus, and changes in ambient light (Fig. 1). Moreover, the users can test their knowledge in quiz mode, where random pupillary abnormalities must be correctly identified and multiple-choice questions about them answered.

We hope Pupil Wizard will provide a valuable addition to traditional teaching of pupil testing. The app is now available on App Store (for iOS devices) and Google Play (for Android devices) and can be downloaded free of charge.
